# Never the Disease “Per Se”

**Published:** 1891-04

**Authors:** A. H. Tompkins

**Affiliations:** Jamaica Plain, Mass.


### NEVER "THE DISEASE PER SE."

EDITORS OF THE HOMŒOPATHIC PHYSICIAN:

I wonder who of us has not forgotten for a moment, as your February correspondent, Dr. J. C. White, seems to have done, that the work of Homœopathy is never that of germicide; that it never doctors diseases "*per se*," but always the individual, with the result, when successful, of making the individual a bad harbor for that disease.

THE HOMŒOPATHIC PHYSICIAN has so much of this teaching on nearly every page that one feels hardly justified in adding to it.

Dr. White says, "My own experience is negative. I have never seen a recovery under homœopathic treatment (of gonorrhœa) in less than six or eight weeks' time. I am satisfied that unaided nature does as well where the subject is in good health."

To this we should have to say, I think, that according to homœopathic philosophy it should be so. That a person in perfect health and strength would either not take gonorrhœa upon exposure, or, taking it, he would without medicine recover in the shortest possible time. The trouble is we do not often have to deal with these theoretical cases of perfect health.

Practically we all have loose joints in our armor of health, latent possibilities of disease, undiscovered weaknesses which are sure to become the skulking-places of imported disease if such a foe is given a chance. The true treatment of a recent case of gonorrhœa is to be determined by no other considerations than those which should govern us in prescribing for a chronic case. In either case the "constitutional dyscrasia" is the true objective-point, not the gonococci. And the dyscrasia, whether namable or otherwise, expresses itself in the symptoms which are not the necessary symptoms of the disease, but, on the contrary, belong to the individual. The ideally healthy man would have no symptoms of this kind in gonorrhœa, he would have the diagnostic symptoms only. Concerning such a case Homœopathy bids us to keep "hands off" and watch only for the appearance of any weak spot in the patient's health which permits the disease *per se* to tarry longer than it should. "Six or eight weeks" is not a long time, providing a benign conclusion waits at the end of that time.

A *healthy* man or woman is an inhospitable host to any wandering gonococcus. The homœopathist's sole duty as a therapeutist in gonorrhœa is to make the subject of it too healthy to harbor the "little beast."

A. H. TOMPKINS.

JAMAICA PLAIN, MASS., February 11th, 1891.

